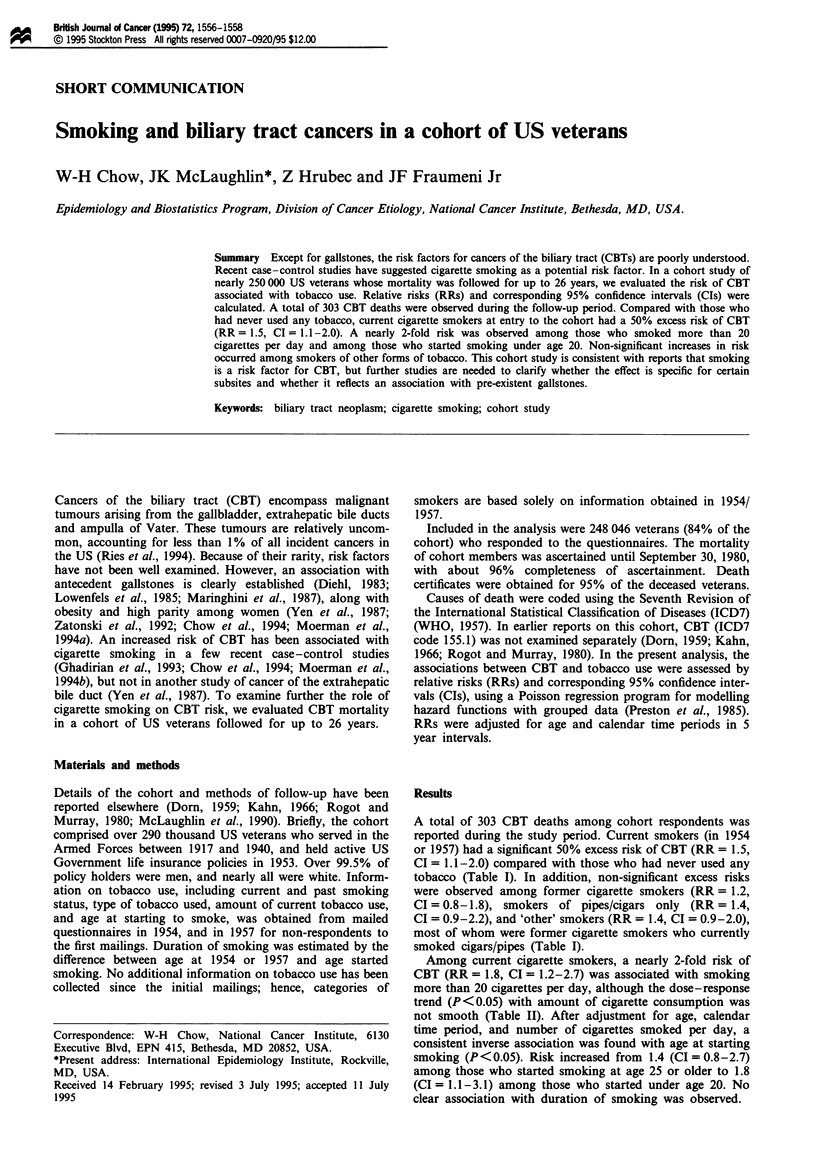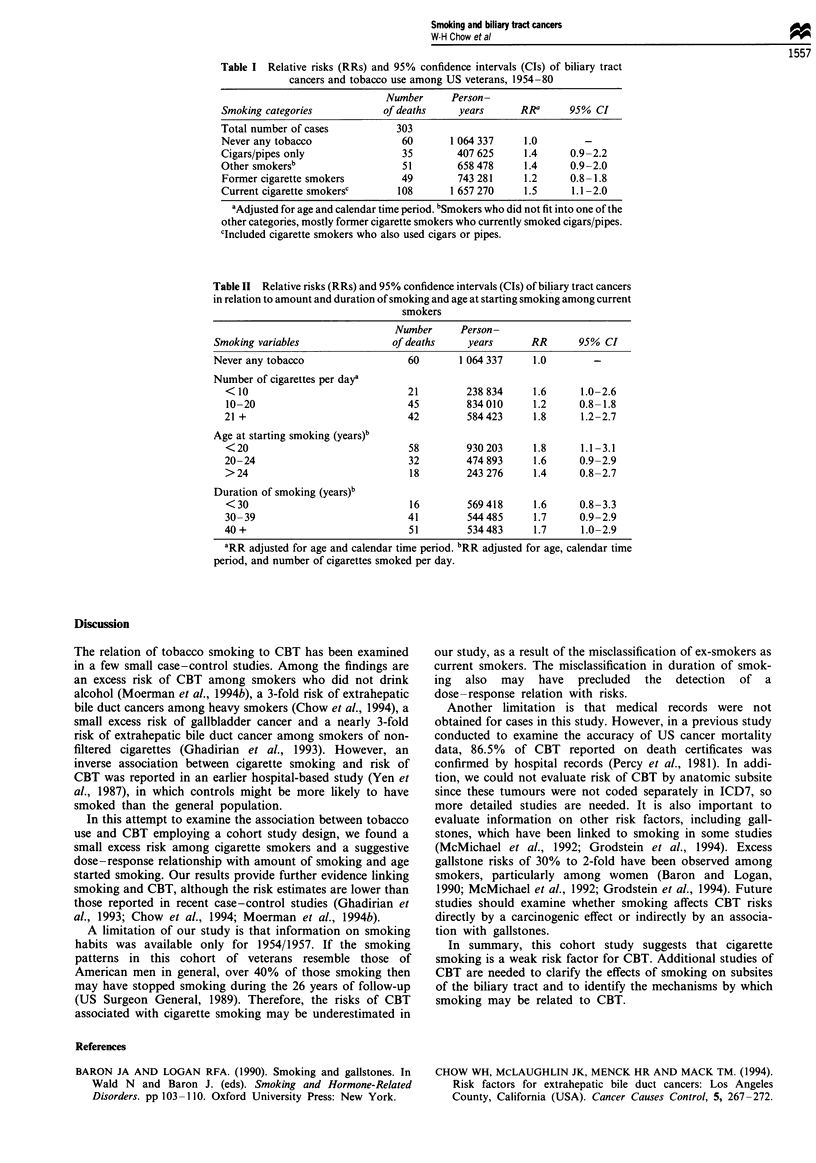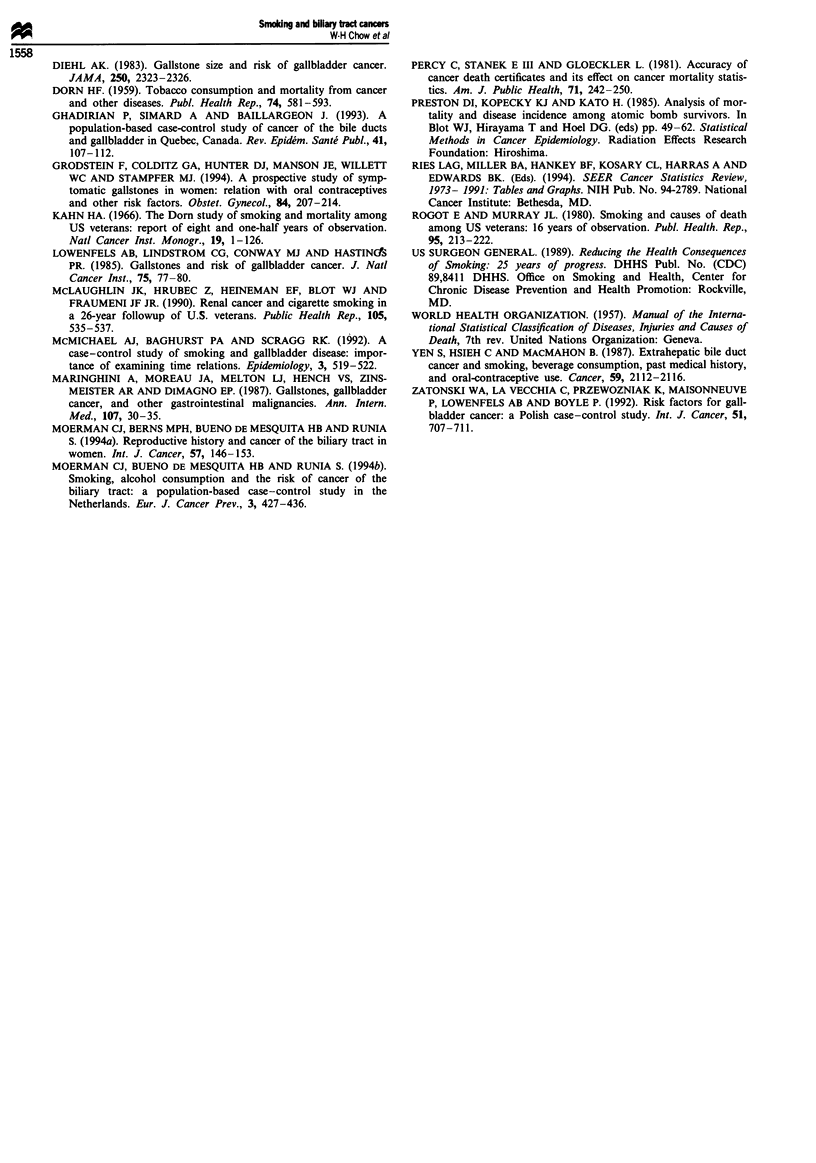# Smoking and biliary tract cancers in a cohort of US veterans.

**DOI:** 10.1038/bjc.1995.547

**Published:** 1995-12

**Authors:** W. H. Chow, J. K. McLaughlin, Z. Hrubec, J. F. Fraumeni

**Affiliations:** Division of Cancer Etiology, National Cancer Institute, Bethesda, MD 20852, USA.

## Abstract

Except for gallstones, the risk factors for cancers of the biliary tract (CBTs) are poorly understood. Recent case-control studies have suggested cigarette smoking as a potential risk factor. In a cohort study of nearly 250,000 US veterans whose mortality was followed for up to 26 years, we evaluated the risk of CBT associated with tobacco use. Relative risks (RRs) and corresponding 95% confidence intervals (CIs) were calculated. A total of 303 CBT deaths were observed during the follow-up period. Compared with those who had never used any tobacco, current cigarette smokers at entry to the cohort had a 50% excess risk of CBT (RR = 1.5, CI = 1.1-2.0). A nearly 2-fold risk was observed among those who smoked more than 20 cigarettes per day and among those who started smoking under age 20. Non-significant increases in risk occurred among smokers of other forms of tobacco. This cohort study is consistent with reports that smoking is a risk factor for CBT, but further studies are needed to clarify whether the effect is specific for certain subsites and whether it reflects an association with pre-existent gallstones.


British Journal of Cancer (1995) 72, 1556-1558
© 1995 Stockton Press All rights reserved 0007-0920/95 $12.00

SHORT COMMUNICATION

### Smoking and biliary tract cancers in a cohort of US veterans

W-H Chow, JK McLaughlin*, Z Hrubec and JF Fraumeni Jr

*Epidemiology and Biostatistics Program, Division of Cancer Etiology, National Cancer Institute, Bethesda, MD, USA.*

**Summary** Except for gallstones, the risk factors for cancers of the biliary tract (CBTs) are poorly understood. Recent case-control studies have suggested cigarette smoking as a potential risk factor. In a cohort study of nearly 250 000 US veterans whose mortality was followed for up to 26 years, we evaluated the risk of CBT associated with tobacco use. Relative risks (RRs) and corresponding 95% confidence intervals (CIs) were calculated. A total of 303 CBT deaths were observed during the follow-up period. Compared with those who had never used any tobacco, current cigarette smokers at entry to the cohort had a 50% excess risk of CBT (RR = 1.5, CI = 1.1-2.0). A nearly 2-fold risk was observed among those who smoked more than 20 cigarettes per day and among those who started smoking under age 20. Non-significant increases in risk occurred among smokers of other forms of tobacco. This cohort study is consistent with reports that smoking is a risk factor for CBT, but further studies are needed to clarify whether the effect is specific for certain subsites and whether it reflects an association with pre-existent gallstones.

**Keywords:** biliary tract neoplasm; cigarette smoking; cohort study

Cancers of the biliary tract (CBT) encompass malignant tumours arising from the gallbladder, extrahepatic bile ducts and ampulla of Vater. These tumours are relatively uncommon, accounting for less than 1% of all incident cancers in the US (Ries *et al.*, 1994). Because of their rarity, risk factors have not been well examined. However, an association with antecedent gallstones is clearly established (Diehl, 1983; Lowenfels *et al.*, 1985; Maringhini *et al.*, 1987), along with obesity and high parity among women (Yen *et al.*, 1987; Zatonski *et al.*, 1992; Chow *et al.*, 1994; Moerman *et al.*, 1994*a*). An increased risk of CBT has been associated with cigarette smoking in a few recent case-control studies (Ghadirian *et al.*, 1993; Chow *et al.*, 1994; Moerman *et al.*, 1994*b*), but not in another study of cancer of the extrahepatic bile duct (Yen *et al.*, 1987). To examine further the role of cigarette smoking on CBT risk, we evaluated CBT mortality in a cohort of US veterans followed for up to 26 years.

### Materials and methods

Details of the cohort and methods of follow-up have been reported elsewhere (Dorn, 1959; Kahn, 1966; Rogot and Murray, 1980; McLaughlin *et al.*, 1990). Briefly, the cohort comprised over 290 thousand US veterans who served in the Armed Forces between 1917 and 1940, and held active US Government life insurance policies in 1953. Over 99.5% of policy holders were men, and nearly all were white. Information on tobacco use, including current and past smoking status, type of tobacco used, amount of current tobacco use, and age at starting to smoke, was obtained from mailed questionnaires in 1954, and in 1957 for non-respondents to the first mailings. Duration of smoking was estimated by the difference between age at 1954 or 1957 and age started smoking. No additional information on tobacco use has been collected since the initial mailings; hence, categories of smokers are based solely on information obtained in 1954/1957.

Included in the analysis were 248 046 veterans (84% of the cohort) who responded to the questionnaires. The mortality of cohort members was ascertained until September 30, 1980, with about 96% completeness of ascertainment. Death certificates were obtained for 95% of the deceased veterans.

Causes of death were coded using the Seventh Revision of the International Statistical Classification of Diseases (ICD7) (WHO, 1957). In earlier reports on this cohort, CBT (ICD7 code 155.1) was not examined separately (Dorn, 1959; Kahn, 1966; Rogot and Murray, 1980). In the present analysis, the associations between CBT and tobacco use were assessed by relative risks (RRs) and corresponding 95% confidence intervals (CIs), using a Poisson regression program for modelling hazard functions with grouped data (Preston *et al.*, 1985). RRs were adjusted for age and calendar time periods in 5 year intervals.

### Results

A total of 303 CBT deaths among cohort respondents was reported during the study period. Current smokers (in 1954 or 1957) had a significant 50% excess risk of CBT (RR = 1.5, CI = 1.1-2.0) compared with those who had never used any tobacco (Table I). In addition, non-significant excess risks were observed among former cigarette smokers (RR = 1.2, CI = 0.8-1.8), smokers of pipes/cigars only (RR = 1.4, CI = 0.9-2.2), and 'other' smokers (RR = 1.4, CI = 0.9-2.0), most of whom were former cigarette smokers who currently smoked cigars/pipes (Table I).

Among current cigarette smokers, a nearly 2-fold risk of CBT (RR = 1.8, CI = 1.2-2.7) was associated with smoking more than 20 cigarettes per day, although the dose-response trend ($P < 0.05$) with amount of cigarette consumption was not smooth (Table II). After adjustment for age, calendar time period, and number of cigarettes smoked per day, a consistent inverse association was found with age at starting smoking ($P < 0.05$). Risk increased from 1.4 (CI = 0.8-2.7) among those who started smoking at age 25 or older to 1.8 (CI = 1.1-3.1) among those who started under age 20. No clear association with duration of smoking was observed.

Correspondence: W-H Chow, National Cancer Institute, 6130 Executive Blvd, EPN 415, Bethesda, MD 20852, USA.
*Present address: International Epidemiology Institute, Rockville, MD, USA.
Received 14 February 1995; revised 3 July 1995; accepted 11 July 1995

**Table I** Relative risks (RRs) and 95% confidence intervals (CIs) of biliary tract cancers and tobacco use among US veterans, 1954–80

| *Smoking categories* | *Number of deaths* | *Person–years* | *RR*[a] | *95% CI* |
|---|---|---|---|---|
| Total number of cases | 303 | | | |
| Never any tobacco | 60 | 1 064 337 | 1.0 | – |
| Cigars/pipes only | 35 | 407 625 | 1.4 | 0.9–2.2 |
| Other smokers[b] | 51 | 658 478 | 1.4 | 0.9–2.0 |
| Former cigarette smokers | 49 | 743 281 | 1.2 | 0.8–1.8 |
| Current cigarette smokers[c] | 108 | 1 657 270 | 1.5 | 1.1–2.0 |

[a]Adjusted for age and calendar time period. [b]Smokers who did not fit into one of the other categories, mostly former cigarette smokers who currently smoked cigars/pipes. [c]Included cigarette smokers who also used cigars or pipes.

**Table II** Relative risks (RRs) and 95% confidence intervals (CIs) of biliary tract cancers in relation to amount and duration of smoking and age at starting smoking among current smokers

| *Smoking variables* | *Number of deaths* | *Person–years* | *RR* | *95% CI* |
|---|---|---|---|---|
| Never any tobacco | 60 | 1 064 337 | 1.0 | – |
| Number of cigarettes per day[a] | | | | |
| <10 | 21 | 238 834 | 1.6 | 1.0–2.6 |
| 10–20 | 45 | 834 010 | 1.2 | 0.8–1.8 |
| 21 + | 42 | 584 423 | 1.8 | 1.2–2.7 |
| Age at starting smoking (years)[b] | | | | |
| <20 | 58 | 930 203 | 1.8 | 1.1–3.1 |
| 20–24 | 32 | 474 893 | 1.6 | 0.9–2.9 |
| >24 | 18 | 243 276 | 1.4 | 0.8–2.7 |
| Duration of smoking (years)[b] | | | | |
| <30 | 16 | 569 418 | 1.6 | 0.8–3.3 |
| 30–39 | 41 | 544 485 | 1.7 | 0.9–2.9 |
| 40 + | 51 | 534 483 | 1.7 | 1.0–2.9 |

[a]RR adjusted for age and calendar time period. [b]RR adjusted for age, calendar time period, and number of cigarettes smoked per day.

### Discussion

The relation of tobacco smoking to CBT has been examined in a few small case–control studies. Among the findings are an excess risk of CBT among smokers who did not drink alcohol (Moerman *et al.*, 1994*b*), a 3-fold risk of extrahepatic bile duct cancers among heavy smokers (Chow *et al.*, 1994), a small excess risk of gallbladder cancer and a nearly 3-fold risk of extrahepatic bile duct cancer among smokers of non-filtered cigarettes (Ghadirian *et al.*, 1993). However, an inverse association between cigarette smoking and risk of CBT was reported in an earlier hospital-based study (Yen *et al.*, 1987), in which controls might be more likely to have smoked than the general population.

In this attempt to examine the association between tobacco use and CBT employing a cohort study design, we found a small excess risk among cigarette smokers and a suggestive dose–response relationship with amount of smoking and age started smoking. Our results provide further evidence linking smoking and CBT, although the risk estimates are lower than those reported in recent case–control studies (Ghadirian *et al.*, 1993; Chow *et al.*, 1994; Moerman *et al.*, 1994*b*).

A limitation of our study is that information on smoking habits was available only for 1954/1957. If the smoking patterns in this cohort of veterans resemble those of American men in general, over 40% of those smoking then may have stopped smoking during the 26 years of follow-up (US Surgeon General, 1989). Therefore, the risks of CBT associated with cigarette smoking may be underestimated in our study, as a result of the misclassification of ex-smokers as current smokers. The misclassification in duration of smoking also may have precluded the detection of a dose–response relation with risks.

Another limitation is that medical records were not obtained for cases in this study. However, in a previous study conducted to examine the accuracy of US cancer mortality data, 86.5% of CBT reported on death certificates was confirmed by hospital records (Percy *et al.*, 1981). In addition, we could not evaluate risk of CBT by anatomic subsite since these tumours were not coded separately in ICD7, so more detailed studies are needed. It is also important to evaluate information on other risk factors, including gallstones, which have been linked to smoking in some studies (McMichael *et al.*, 1992; Grodstein *et al.*, 1994). Excess gallstone risks of 30% to 2-fold have been observed among smokers, particularly among women (Baron and Logan, 1990; McMichael *et al.*, 1992; Grodstein *et al.*, 1994). Future studies should examine whether smoking affects CBT risks directly by a carcinogenic effect or indirectly by an association with gallstones.

In summary, this cohort study suggests that cigarette smoking is a weak risk factor for CBT. Additional studies of CBT are needed to clarify the effects of smoking on subsites of the biliary tract and to identify the mechanisms by which smoking may be related to CBT.

### References

BARON JA AND LOGAN RFA. (1990). Smoking and gallstones. In Wald N and Baron J. (eds). *Smoking and Hormone-Related Disorders*. pp 103–110. Oxford University Press: New York.

CHOW WH, MCLAUGHLIN JK, MENCK HR AND MACK TM. (1994). Risk factors for extrahepatic bile duct cancers: Los Angeles County, California (USA). *Cancer Causes Control*, **5**, 267–272.

DIEHL AK. (1983). Gallstone size and risk of gallbladder cancer. *JAMA*, **250**, 2323–2326.

DORN HF. (1959). Tobacco consumption and mortality from cancer and other diseases. *Publ. Health Rep.*, **74**, 581–593.

GHADIRIAN P, SIMARD A AND BAILLARGEON J. (1993). A population-based case-control study of cancer of the bile ducts and gallbladder in Quebec, Canada. *Rev. Epidém. Santé Publ.*, **41**, 107–112.

GRODSTEIN F, COLDITZ GA, HUNTER DJ, MANSON JE, WILLETT WC AND STAMPFER MJ. (1994). A prospective study of symptomatic gallstones in women: relation with oral contraceptives and other risk factors. *Obstet. Gynecol.*, **84**, 207–214.

KAHN HA. (1966). The Dorn study of smoking and mortality among US veterans: report of eight and one-half years of observation. *Natl Cancer Inst. Monogr.*, **19**, 1–126.

LOWENFELS AB, LINDSTROM CG, CONWAY MJ AND HASTINGS PR. (1985). Gallstones and risk of gallbladder cancer. *J. Natl Cancer Inst.*, **75**, 77–80.

MCLAUGHLIN JK, HRUBEC Z, HEINEMAN EF, BLOT WJ AND FRAUMENI JF JR. (1990). Renal cancer and cigarette smoking in a 26-year followup of U.S. veterans. *Public Health Rep.*, **105**, 535–537.

MCMICHAEL AJ, BAGHURST PA AND SCRAGG RK. (1992). A case–control study of smoking and gallbladder disease: importance of examining time relations. *Epidemiology*, **3**, 519–522.

MARINGHINI A, MOREAU JA, MELTON LJ, HENCH VS, ZINSMEISTER AR AND DIMAGNO EP. (1987). Gallstones, gallbladder cancer, and other gastrointestinal malignancies. *Ann. Intern. Med.*, **107**, 30–35.

MOERMAN CJ, BERNS MPH, BUENO DE MESQUITA HB AND RUNIA S. (1994*a*). Reproductive history and cancer of the biliary tract in women. *Int. J. Cancer*, **57**, 146–153.

MOERMAN CJ, BUENO DE MESQUITA HB AND RUNIA S. (1994*b*). Smoking, alcohol consumption and the risk of cancer of the biliary tract: a population-based case–control study in the Netherlands. *Eur. J. Cancer Prev.*, **3**, 427–436.

PERCY C, STANEK E III AND GLOECKLER L. (1981). Accuracy of cancer death certificates and its effect on cancer mortality statistics. *Am. J. Public Health*, **71**, 242–250.

PRESTON DI, KOPECKY KJ AND KATO H. (1985). Analysis of mortality and disease incidence among atomic bomb survivors. In Blot WJ, Hirayama T and Hoel DG. (eds) pp. 49–62. *Statistical Methods in Cancer Epidemiology*. Radiation Effects Research Foundation: Hiroshima.

RIES LAG, MILLER BA, HANKEY BF, KOSARY CL, HARRAS A AND EDWARDS BK. (Eds). (1994). *SEER Cancer Statistics Review, 1973– 1991: Tables and Graphs*. NIH Pub. No. 94-2789. National Cancer Institute: Bethesda, MD.

ROGOT E AND MURRAY JL. (1980). Smoking and causes of death among US veterans: 16 years of observation. *Publ. Health. Rep.*, **95**, 213–222.

US SURGEON GENERAL. (1989). *Reducing the Health Consequences of Smoking: 25 years of progress*. DHHS Publ. No. (CDC) 89,8411 DHHS. Office on Smoking and Health, Center for Chronic Disease Prevention and Health Promotion: Rockville, MD.

WORLD HEALTH ORGANIZATION. (1957). *Manual of the International Statistical Classification of Diseases, Injuries and Causes of Death*, 7th rev. United Nations Organization: Geneva.

YEN S, HSIEH C AND MACMAHON B. (1987). Extrahepatic bile duct cancer and smoking, beverage consumption, past medical history, and oral-contraceptive use. *Cancer*, **59**, 2112–2116.

ZATONSKI WA, LA VECCHIA C, PRZEWOZNIAK K, MAISONNEUVE P, LOWENFELS AB AND BOYLE P. (1992). Risk factors for gallbladder cancer: a Polish case–control study. *Int. J. Cancer*, **51**, 707–711.